# Bromocriptine and Colesevelam Hydrochloride: Novel Therapies for Type II Diabetes Mellitus

**DOI:** 10.7759/cureus.50138

**Published:** 2023-12-07

**Authors:** Lenise G Soileau, Angela Nguyen, Aarthi Senthil, Jolie A Boullion, Norris C Talbot, Shahab Ahmadzadeh, Sahar Shekoohi, Alan D Kaye, Giustino Varrassi

**Affiliations:** 1 School of Medicine, Louisiana State University Health Sciences Center, Shreveport, USA; 2 Department of Anesthesiology, Louisiana State University Health Sciences Center, Shreveport, USA; 3 Department of Pain Medicine, Paolo Procacci Foundation, Rome, ITA

**Keywords:** cardiovascular disease, drug adherence, bile acid sequestrant, dopamine agonists, insulin sensitivity, type ii diabetes, colesevelam hydrochloride, bromocriptine

## Abstract

The increasing prevalence of type II diabetes mellitus (T2DM) is a worldwide healthcare concern. Over the years, our understanding of T2DM has grown considerably in uncovering the pathophysiology of the disease and, in turn, understanding how improved treatment methods can be used to slow disease progression. Some long-term complications that are responsible for most T2DM mortalities include cardiovascular disease, neurological decline, and renal failure. In treating T2DM, it is important that not only glycemic control be obtained but also control of associated complications. Bromocriptine and colesevelam hydrochloride have both been approved by the Food and Drug Administration (FDA) to treat T2DM but are not readily used in practice. These medications are known to treat glycemic dysregulation via unconventional mechanisms, which might contribute to their potential to provide protection against common diabetic complications such as cardiovascular disease. In order to ensure that these overlooked medications become more readily used, it is vital that more research be performed to further elucidate their efficacy in a clinical setting. Future studies should continue to provide clinicians a better understanding of the role these medications have on the treatment of T2DM such as their ability to be used in combination with other commonly used T2DM medications or as monotherapies.

## Introduction and background

Type II diabetes mellitus (T2DM) is a chronic and incurable metabolic disorder that has long been considered among some of the most costly and debilitating diseases in healthcare, both regionally and worldwide. This chronic disease is currently documented as the seventh leading cause of death in the United States as a result of many comorbidities and complications associated with uncontrolled cases [1]. Such comorbidities include kidney failure, obesity, coronary artery disease, peripheral vascular disease, hypertension, stroke, and amputations. Currently, rates of T2DM are on the rise, with the increased prevalence of cases among youth (>20 years) being one of the most alarming trends. A recent study projecting this rise in prevalence estimated a 69% increase (28,000-48,000) in yearly diagnosed youth from 2017 to 2060, assuming current trends remain unchanged [2]. This growth increases concern since the risk of diabetes complications is higher for those diagnosed at a younger age, driving healthcare costs even higher. Coordinated efforts must be made to educate the public and provide more affordable and effective treatment options [3].

While there are many attempts to combat the growing prevalence of T2DM, one of the more important initiatives is the research and development of novel treatment options for lowering blood glucose. Currently, there are a wide variety of medications available for treating T2DM, but adverse side effects, low efficacy, and financial burdens of treatments hinder the impact these medications could have in treating this disease by decreasing patient adherence. The decline in adherence to diabetic treatments can accelerate long-term complications often associated with T2DM. In a study conducted from July to December 2020, researchers found that out of 483 participants, only 61% (n=305) were adherent, while 36.9% (n=178) were at some point non-adherent to medications [4]. Allowing patients to engage in their care by providing an individualized treatment plan suited to patient preferences, such as choices of administration routes (injection vs. oral), treatment goals, and side effect considerations, is an effective way to increase adherence. These patient-centered approaches require the development of more treatment options that can better align with specific patient needs [5,6].

Developing medications that can not only reduce hyperglycemia but also simultaneously treat other associated complications and risk factors of T2DM is another important initiative. Currently, many emerging drugs being studied are targeting metabolic pathways that either lead to weight loss or mitigate T2DM complications such as cardiovascular disease and hyperlipidemia [7]. Other future novel targets such as vaspin, Metrnl, and fetuin-A are being explored as safer and more effective mechanisms for regulating glucose when compared to current conventional treatment options [8]. Bromocriptine (BC) (Cycloset, VeroScience, Tiverton, Rhode Island, United States) and colesevelam hydrochloride (Welchol, Alkem Laboratories, Mumbai, India) are two underused medications that have been Food and Drug Administration (FDA)-approved to treat T2DM for over a decade. BC is a sympatholytic dopamine D2 receptor agonist that acts centrally, allowing it to reduce plasma glucose, triglycerides, and free fatty acids (FFA) [9]. Colesevelam hydrochloride acts as a bile acid sequestrant (BAS), which accelerates plasma low-density lipoprotein (LDL) clearance and has the ability to reduce both blood glucose and cholesterol levels [10]. This review aims to provide an in-depth understanding of the mechanisms and recent clinical findings related to the use of BC and colesevelam hydrochloride for the treatment of T2DM.

## Review

Epidemiology, pathophysiology, and complications of T2DM

Epidemiology of T2DM

T2DM is a growing global health concern, affecting over 400 million people worldwide. It is found that genetics and the environment play a role in its epidemiology. It is also commonly found in about 80% of patients who live in low- to middle-income areas. In 2015, according to the International Diabetes Federation (IDF), about 10% of the American population have diabetes, with nearly 7 million people undiagnosed [11]. Additionally, in the population of individuals above 65 years old, approximately 25% were diagnosed with diabetes [11]. In 2019, there were approximately 4.2 million deaths associated with complications related to uncontrolled cases [12]. These staggering statistics highlight the growing need for continued progress in the early diagnosis and treatment of T2DM.

Pathophysiology of T2DM

T2DM is characterized by elevated blood glucose levels caused by a dysregulation in insulin sensitivity and secretion. Initially, T2DM begins as insulin resistance, characterized by a diminished insulin response leading to impaired action in metabolically active organs and tissues such as the liver, muscle, and adipose. When tissues are insulin-resistant, glucose is unable to enter cells and remains in the blood, inducing hyperglycemia. The pancreas counters this high blood glucose by increasing insulin production to maintain glucose homeostasis [11]. However, over time, the dysfunctional beta cells are unable to keep up with this increased insulin demand, resulting in a decline in insulin production, further perpetuating hyperglycemia [13]. Furthermore, clinical findings have suggested several other potential factors that lead to the development of T2DM. Many patients diagnosed with T2DM have a higher fat percentage, predominantly in the abdominal region and often surrounding the liver. This adipose tissue can induce the expression of inflammatory chemokines and cytokines, which can further impair proper insulin function. Additional data suggests that peripheral exogenous dopamine plays a role in the regulation of insulin signaling pathways and glucose uptake in insulin-sensitive tissues by influencing dopamine receptors. This connection was shown in one study when mice that lacked dopamine 2 receptors had abnormal insulin secretion and were glucose-intolerant [14,15]. Furthermore, a dopamine receptor blockade in mice resulted in adverse metabolic profiles such as hyperinsulinemia, weight gain, and glucose intolerance [16]. Dopamine's role in insulin regulation is an important finding as a potential target for future and present pharmaceutical treatments. The absence of physical exercise is associated with dyslipidemia and hypertension, which have been known to accelerate the pathogenesis of insulin resistance, leading to T2DM. Other rare but certainly important causes such as the disruption with incretin biology resulting from decreased synthesis of glucagon-like peptide-1 (GLP-1) or incretin resistance along with adipokine dysregulation, glucagonomas, inflammation, increased renal glucose reabsorption, and abnormalities in gut microbiota may also play a role in the development of T2DM [13].

Complications of T2DM

Diagnosing T2DM in prediabetic stages or before the disease becomes chronic is of great importance to successful patient outcomes. In early disease stages, symptoms are generally mild, which often leads to delayed diagnosis. Typically, patients who are diagnosed with T2DM present with a lack of energy, fatigue, polydipsia, and polyuria. Delayed wound healing and increased infection rate may be associated with immunocompromising T2DM complications. In more chronic and uncontrolled T2DM, patients experience neuronal damage, which includes blurred vision as a result of diabetic retinopathy and tingling or numbness in distal extremities, known as diabetic neuropathy [17]. Additionally, other manifestations of insulin resistance include non-alcoholic fatty liver disease, nephropathy, essential hypertension, obesity, dyslipidemia, ovarian hyperandrogenism, and premature adrenarche [17,18]. The vast complications of long-term T2DM are crippling and often lead to poor prognosis. As such, new medications are commonly seeking to specifically target these complications through mechanisms that lead to weight loss, lowering blood lipid levels, and regulating key endocrine pathways.

Current treatments for T2DM

Current treatment protocols for the glycemic regulation of T2DM include lifestyle changes and pharmaceutical therapies. Some of the earliest attempts to treat T2DM seek to incorporate healthy lifestyle choices, including implementing physical activity, weight loss management, and healthy dietary intake. Such practices are still some of the most fundamental treatment approaches beyond pharmaceutical options, with proven benefits in reducing the risk of associated neural and vascular complications [19]. Pharmaceutical options are among the most readily used forms of diabetes management. The recent expansion of knowledge on the pathophysiology of T2DM has led to the exponential development of approved pharmaceutical treatment options, including insulin and non-insulin medications. While insulin was once the drug of choice for all forms of diabetes, the synthesis of non-insulin medications has made the use of insulin as a first- or even second-line treatment option nearly obsolete.

Commonly used non-insulin pharmaceutical therapies and the growing need for new treatment options

Most newly designed drug interventions for treating T2DM are classified as "non-insulin" medications with mechanisms that either increase insulin secretion by the pancreas or promote glucose excretion. Four of the most commonly used classes of non-insulin drugs are biguanides (metformin), dipeptidyl peptidase-4 (DPP-4) inhibitors, GLP-1 agonists, and sodium glucose co-transporter-2 (SGLT2) inhibitors. Characteristics of these classes, including the level of A1C% decrease, advantages, disadvantages, and general cost, are depicted in Table 1 [20].

**Table 1:** Informatics table depicting various important findings among common non-insulin medications. BID: twice (two times) a day; QW: once weekly; NAFLD: nonalcoholic fatty liver disease; DKA: diabetic ketoacidosis Source: [20]

	Medication names	A1C% decrease	Advantages	Disadvantages	Cost
Metformin	Metformin (2000 mg)	1.01	No hypoglycemia, once-a-day administration, no weight gain (possible weight loss), may decrease cardiovascular disease	Gastrointestinal side effects, B12 deficiency, lactic acidosis (very rare), need to monitor renal function	Low
	Metformin (2550 mg)	1.09			
Dipeptidyl peptidase-4 inhibitors	Alogliptin (12.5 mg)	0.58	No hypoglycemia, weight neutral, decrease postprandial glucose, once-a-day administration, well tolerated, decrease blood pressure	Pancreatic disease/heart failure (saxagliptin/alogliptin), arthritis bullous pemphigoid, modest glycemic lowering	High
	Alogliptin (25 mg)	0.66			
	Linagliptin (5 mg)	0.59			
	Saxagliptin (2.5 mg)	0.59			
	Saxagliptin (5 mg)	0.67			
	Sitagliptin (100 mg)	0.72			
Glucagon-like peptide-1 agonists	Exenatide (10 ug BID)	1.18	Weight loss, no hypoglycemia, reduce cardiovascular disease (liraglutide, semaglutide, dulaglutide), improve NAFLD, possible once-a-week therapy, decrease albuminuria, decrease postprandial glucose	Gastrointestinal side effects, requires injection, gall bladder disease, possible pancreatitis and thyroid cancer?	High
	Exenatide (2 mg QW)	1.36			
	Liraglutide (0.6 mg)	1.16			
	Liraglutide (1.2 mg)	1.14			
	Lixisenatide (10 ug)	1.13			
	Lixisenatide (20 ug)	1.25			
Sodium glucose co-transporter-2 inhibitors	Canagliflozin (100 mg)	0.84	Weight loss, no hypoglycemia, decrease heart failure, decrease renal dysfunction, once-a-day administration, decrease blood pressure	Urinary tract infections, genital mycotic infections, increased low-density lipoprotein (small increase), increased risk of DKA, postural hypotension/volume depletion	High
	Canagliflozin (300 mg)	1.01			
	Dapagliflozin (5 mg)	0.65			
	Dapagliflozin (10 mg)	0.73			
	Empagliflozin (10 mg)	0.69			
	Empagliflozin (25 mg)	0.77			

Despite the numerous available pharmaceutical options for treating T2DM, there is still a growing need for the development of non-insulin medications that address treatment burdens. It has been reported that at least 45% of patients prescribed pharmaceutical therapies fail to achieve adequate glycemic control due to poor medication adherence [21]. Many known modifiable factors have been shown to lead to poor medication adherence, including side effects, treatment complexity, inconvenience, and cost-effectiveness. Among antidiabetic medications, the most well-known associated side effects include hypoglycemia and gastrointestinal (GI) issues. A single hypoglycemic event can cause fear in some patients, leading to medication non-adherence or the tendency for patients to be pressured to keep blood glucose levels high as a preventative measure [22,23]. In a cross-sectional study, it was found that patients diagnosed with T2DM who had poor medication adherence to metformin and sulfonylurea agents reported moderate or worse symptoms of hypoglycemia compared to those who had higher medication adherence [24]. GLP-1 agonists, which stimulate glucose-dependent insulin release while providing an added benefit in weight loss, are often associated with GI issues such as nausea, vomiting, diarrhea, and pancreatitis. A retrospective analysis recently found discrepancies in patient-provider communication about the rationale for patients discontinuing GLP agonists. While GI complaints were noted as a primary concern among both parties, the lack of blood glucose control and weight loss were other factors not effectively communicated, which potentially led to decreased adherence [25]. Overall, side effects like hypoglycemia and GI problems exemplify the need for new diabetic therapies. An emphasis on fine-tuning patient-physician communication when discussing medication disadvantages could help alleviate some current adherence challenges.

Other potential modifiable factors for medication adherence include treatment complexity, convenience, and cost. Treatments that are perceived as more difficult and burdensome also contribute to poor medication adherence. In several reviews, it has been found that patients with chronic T2DM had poor medication adherence rates for a prescription regimen that required more than once daily dosing (79-94% once daily vs. 38-67% three times daily; P<0.05) [26,27]. The method of administration of antidiabetic medications is another burden for many patients and often leads to decreased adherence. It has been found that one of the reasons patients discontinued the GLP-1 agonists was related to its route of administration, which is less preferred by some when compared to oral medications [25]. Lastly, many new antidiabetic medications are unaffordable, making them less accessible as treatment options. In a retrospective analysis, data showed that T2DM patients with a low-income subsidy for Medicare Part D had a lower out-of-pocket cost and higher medication adherence compared to those who did not receive the subsidy [28]. Though GLP-1 agonists have shown promising long-term cost-effectiveness by their ability to significantly lower A1C levels, many individuals are unable to afford them. This financial burden leads many to be prescribed potentially less cost-effective treatments such as sulfonylureas and pioglitazone, which have been shown to have more severe side effect burdens [29]. Overall, the studies above illustrate the continued need for new antidiabetic therapies, which are less costly and burdensome for patients. Providing more abundant treatment options will allow patients to have a more active role in determining the medication suitable to fit a patient's needs, leading to more efficacious treatment outcomes through enhanced adherence rates.

BC as a novel option for treating T2DM

Synthesized from the fungal parasite ergot, BC is an ergot alkaloid derivative that acts as a D2 dopamine receptor agonist. In the clinical setting, BC is mainly used to treat patients with Parkinson's disease, acromegaly, and hyperprolactinemia. However, more attention is drawn to BC's involvement with insulin resistance. This drug was approved by the FDA in May of 2009 as a T2DM therapy in adjunct to diet and exercise [30,31]. Morning doses of BC increase dopamine levels, which in turn decreases sympathetic nervous system activity, leading to decreased hepatic glucose production, gluconeogenesis, and lipogenesis [9,32,33]. These sympathetic effects may also contribute to its role in reducing blood pressure [20]. 

The exact mechanism of action of BC is not fully understood; however, peripheral dopamine's involvement in insulin-sensitive tissues is one part of the mechanism. Pancreatic beta cells have dopamine D2-like receptors (D2R and D3R) that can work with insulin receptors on the same tissue [34]. In a 2021 experimental trial, the effects of dopamine on glucose uptake in the liver, soleus muscle, and white and brown adipose tissues were evaluated in mice. With dopamine delivered intravenously before glucose orally, the glucose uptake significantly increased compared to the controls in all sampled tissues except the mesenteric white adipose tissue [35]. This result indicates that BC treatment increases GLUT4 transporters and insulin receptor expression, aiding in glucose uptake [36]. 

BC, in conjunction with sitagliptin, a DPP-4 inhibitor, has shown increased insulin secretion aiding T2DM management. Independently, sitagliptin inhibits the degradation of incretins, GLP-1, and glucose-dependent insulinotropic peptide by inhibiting DPP-4 (a GLP-1-inactivating peptide), which serve to initiate the secretion of postprandial insulin [32,37]. Considering a few clinical trials that investigated the efficacy of sitagliptin, it can be used as a relatively safe antidiabetic treatment that works independently but shows greater effectiveness when administered as an adjunct therapy with BC [38]. In a 2018 experimental study, induced diabetic rats were treated with BC, sitagliptin 10 mg/kg orally (SG10), sitagliptin 20 mg/kg orally (SG20), and a combination of SG10+BC over two weeks. Levels of serum glucose, serum fructosamine, serum insulin, and Homeostasis Model Assessment Index for Insulin Resistance (HOMA-IR) decreased with all four treatment protocols. Among the BC+SG10 treatment group, findings related to treatment benefits were similar to the higher-dose SG20 treatment group but greater than the individual groups alone. There was an increase in serum GLP-1, phosphorylated insulin receptor (p-IR) at tyrosine residues 1162 and 1163, phosphorylated protein kinase B (p-AKT) at serine 473, GLUT4, and improved lipid panels (triglycerides, cholesterol, and LDL-C) as compared to the diabetic control rats with the BC+SG10 treatment yielding the greatest difference in values [32]. Furthermore, the peroxisome proliferator-activated receptor-gamma (PPAR-γ) pathway is involved with upregulating glucose transporters and thereby increases glucose entry into muscle and adipose tissues. For T2DM patients, the PPAR-γ pathway role is diminished, leading to insulin insensitivity [39,40]. Additionally, T2DM patients have a high activity of the Janus kinase (JAK)/signal transducer and activator of transcription proteins (STAT) pathway, which induces the inflammation of endothelial cells via interleukin-6 [38]. The BC+SG10 treatment results indicated that both pathways were involved. This trial revealed that sitagliptin+BC does not only function through increasing GLP-1 but has other confounding mechanisms that contribute to a more comprehensive treatment for T2DM.

As previously mentioned, the impact that BC has on the diminished activation of the sympathetic nervous system may provide insight into its ability to decrease cardiovascular risk potentially. This could be directly related to its regulatory effects on blood pressure through decreased vascular tone [33,20]. However, other studies suggest that better cardiovascular outcomes might be caused by BC's association with modest weight loss [41,42]. Future studies should be performed to better elucidate the mechanisms that enable BC to provide glycemic control, along with its potential role in protection against cardiovascular risk. Important characteristics of BC, including advantages, disadvantages, and general cost, are depicted in Table 2 [20].

**Table 2:** Informatics table depicting important findings related to bromocriptine. Source: [20]

	Advantages	Disadvantages	Cost
Bromocriptine	Modest decrease in glycated hemoglobin levels (HbA1C), decreased triglycerides, once-a-day dosing, possible cardiovascular benefits, decreased blood pressure, slight weight loss, no hypoglycemia	Need to titrate dose, discontinuation due to gastrointestinal side effects	High

Colesevelam hydrochloride as a novel option for treating T2DM

Colesevelam hydrochloride is another potential adjunct treatment option for patients with T2DM. This treatment was originally used to treat hyperlipidemia by lowering LDL-C but became FDA-approved in 2008 to be used in combination with insulin, metformin, or sulfonylureas to treat T2DM. Functioning as a BAS, colesevelam forms bile acid complexes with its extensive hydrophobic side chain network, preventing further bile acid from being reabsorbed. This drug property activates the conversion of cholesterol into bile acid, thereby lowering intracellular cholesterol levels [43]. The mechanism of colesevelam for treating T2DM is not fully known, but there is increasing evidence of its involvement with activating GLP-1 and decreasing serum glucose levels. Colesevelam does not influence insulin secretion, glucose absorption, or postprandial insulin levels, indicating its lack of effect on insulin resistance [43]. By increasing cholecystokinin levels and activating TGR5, a G-protein-coupled receptor for bile acids, GLP-1 levels increase, and gastric emptying is slowed. These effects suppress hepatic glycogenolysis and reduce serum glucose levels [30,31,43]. When surveying colesevelam as an adjunct therapy in mice models, it was found that the combination of colesevelam+metformin and colesevelam+sulphonylureas decreased the HbA1C levels by 0.54% (P=.001) [30]. 

Compared to other BAS treatments, earlier treatments had high discontinuation rates and poor adherence. These findings are partly due to severe GI side effects such as bloating, gas, constipation, difficult dosing schedules, and the unpleasant taste of the medication. Colesevelam, on the other hand, is more potent, so less medication is required to achieve a similar efficacy as its previous counterparts. It has also been reported to have fewer side effects, but trials regarding direct comparisons of colesevelam versus other likewise treatments are very limited [44]. Important characteristics of colesevelam, including advantages, disadvantages, and general cost, are depicted in Table 3 [20].

**Table 3:** Informatics table depicting important findings related to colesevelam. LDL-C: low-density lipoprotein cholesterol Source: [20]

	Advantages	Disadvantages	Cost
Colesevelam	Modest decrease in glycated hemoglobin (HbA1C), lowers LDL-C, minimal systemic effects, once-a-day administration possible, no hypoglycemia, neutral weight effect	Increases triglyceride levels particularly if already high, gastrointestinal side effects, inhibits the absorption of other drugs	High

Clinical studies with BC

BC-QR, the quick-release formulation of the dopamine D2 receptor agonist BC, improves glycemic control in T2DM patients. BC-QR is a circadian-timed therapy given within two hours of waking to mimic the natural spike in central dopaminergic activity depleted in T2DM patients with insulin resistance [45,46]. In a 2014 randomized open-label study with 74 enrolled patients, BC combined with diabetes medication metformin significantly decreased fasting plasma glucose and postprandial plasma glucose levels in an increasing dose-dependent manner (0.8 mg and 1.6 mg/day) at four, eight, and 12 weeks compared to baseline [47]. Although HbA1C levels decreased for all treatment groups, metformin alone (1000 mg/day), BC (0.8 mg/day)+metformin, and BC (1.6 mg/day)+metformin at 12 weeks, the treatment groups with BC decreased significantly compared to the metformin alone group. Only 8% of patients in the 0.8 mg/day BC group and 12% in the 1.6 mg/day BC group withdrew due to mild to moderate adverse effects (AE) (nausea, vomiting, and dizziness). No life-threatening AE was reported [47].

A 2015 pilot study evaluated the use of BC-QR in seven 30-65-year-old T2DM patients on metformin (1-2 g/day) and high-dose basal-bolus insulin (total daily insulin dose (TDID)≥65 U/day) with HbA1C between 7.5% and 12.0% for 24 weeks [23]. Patients added the BC-QR therapy within two hours of waking, starting the first week with 0.8 mg and increasing the dose by an additional 0.8 mg tablet each following week until a maximum tolerated dose of two to six tablets (1.6-4.8 mg) was reached. Five patients achieved a maximum dose of 4.8 mg/day, and two patients continued the study at a maximum of 1.6 mg/day due to AE of nausea and headaches. Adding BC-QR resulted in a statistically significant decline in HbA1C levels by 1.76% and TDID by 27%. Although limited by a small sample size, the pilot study found a decrease in the TDID requirement and an improvement in glycemic control and meal tolerance in T2DM subjects with the addition of BC-QR therapy [23]. A similar 2017 study of 60 T2DM subjects with inadequately controlled insulin on metformin and basal-bolus insulin found a 0.73% and 1.13% reduction in HbA1C relative to baseline and placebo (p<0.001), respectively, with the administration of BC-QR [46]. This study included more subjects than the 2015 pilot study but was limited in duration to 12 weeks [47]. These clinical findings, as indicated in Table 4, demonstrate that BC-QR therapy, when added to other common T2DM medication regimens, has great efficacy at improving glycemic control. Although BC is attributed to its effects in remodeling dopaminergic systems in the brain, its role in peripheral dopaminergic systems is unclear [36,48]. In a 2021 study, insulin-resistant patients displayed decreased expression of certain dopamine receptor genes that correlated with adipocyte metabolic function such as dopamine receptor D1. BC was found to restore the function of this depleted dopamine receptor in white adipose tissue and the liver. Thus, it is likely that BC also modulates peripheral dopaminergic systems to restore increased insulin sensitivity and catabolic metabolism [36]. Further evidence is needed to understand the complete pathophysiological action of BC. Overall, BC-QR is safe and efficacious in improving glycemic control and sensitivity in numerous clinical studies.

**Table 4:** Baseline vs. end-of-study values for study groups adding bromocriptine therapy along with other commonly used anti-glycemic medications. Normal values for the average glycated hemoglobin levels (HbA1C (%)) and FPG are <5.7% and 80-90 mg/dl, respectively. FPG: fasting plasma glucose Note: All study groups received bromocriptine therapy in combination with metformin. In Roe et al. and Chamarthi and Cincotta, bromocriptine groups additionally received high-dose basal-bolus insulin. Ghosh et al. had two study groups receiving bromocriptine 0.8 mg/day ("1") and 1.6 mg/day ("2").

	Average glycated hemoglobin levels (HbA1C(%))		Average total daily insulin dose (units)		FPG (mg/dL)		Total study length (weeks)
	Baseline	Final	Baseline	Final	Baseline	Final	
Ghosh et al. [47]	7.92±0.51^1^; 7.89±0.62^2^	7.03±0.61^1^; 6.6±0.65^2^; P<0.05	-	-	165.9±8.9^1^; 167.8±7.3^2^	105.6±7.8^1^; 89.9±9.1^2^; P<0.05	12
Roe et al. [23]	9.74±0.56	7.98±0.36; P=0.01	199±33	147±31; P=0.009	243±31	229±16	24
Chamarthi and Cincotta [46]	8.42±0.17	7.47±0.23; P<0.001	119.3±7.8	118.2±8.4	157.9±11.2	146.9±8.7	12

Clinical studies with colesevelam hydrochloride

Colesevelam was originally developed for reducing cholesterol levels but was later found to enhance glucose tolerance in T2DM patients [48]. Colesevelam is a BAS, a type of drug that binds bile acids and increases their fecal loss. The connection between this action and how it improves glucose metabolism in humans is still unclear and being investigated. Some animal studies have suggested an increase in GLP-1 is linked to the action of BAS like colesevelam in glucose control.

In a randomized, double-blind study, 10 healthy patients and nine T2DM patients on current treatment with metformin therapy alone received chenodeoxycholic acid (CDCA), colesevelam, CDCA+colesevelam, or placebo by nasogastric feeding tube. On four separate days, participants were asked to fast overnight for 10 hours, to refrain from exercise the day before and the day of the experiment, and to abstain from metformin use one week prior to an experimental day. Blood was sampled at baseline and specific increments up to 180 minutes after administration to evaluate GLP-1, glucose-dependent insulinotropic polypeptide, glucose, insulin, C-peptide, glucagon, cholecystokinin, and gastrin. Their data found that colesevelam did not affect GLP-1 secretion nor plasma glucose, insulin, or C-peptide in human subjects [48].

In an animal study searching for the link between the reduction of cholesterol and glucose by colesevelam and the role of hepatic microRNAs in pathological metabolic processes, no significant differences in GLP-1 levels after colesevelam treatment were found for fasting or post-glucose groups [49]. However, colesevelam was found to lower systemic glucose levels in Zucker diabetic fatty rats and db/db mice. Additionally, investigated by high-throughput sequencing and real-time polymerase chain reaction (PCR), a cholesterol-sensitive hepatic microRNA miR-96/182/183 was significantly elevated in the livers of rats treated with colesevelam compared to the control. The study also demonstrated that this microRNA directly diminishes the expression of a gene connected to hepatic lipid metabolism, known as MED1, indicating that colesevelam works through cholesterol-related gene regulation to enhance glycemic control [49].

LDL-C reduction is recommended by 2018 American Heart Association (AHA) guidelines for patients at high risk for coronary artery disease, including patients with T2DM [50,51]. In a 2020 study lasting 24 weeks, 200 subjects on a stable statin and diabetic medications were randomized to either open-labeled colesevelam or ezetimibe (both second-line LDL-C therapies) with a significant decline in LDL-C levels at the end of the study period (14% and 23.2%, respectively) [51]. Patients treated with colesevelam (26.8%) reached the study's goal HbA1C level (≤7.0%), significantly more than those treated with ezetimibe (14.7%, P=.04). However, more participants in the colesevelam group experienced at least one AE (usually GI problems) compared to the ezetimibe group (20.2% vs. 7.2%, respectively, P= .009). These findings can help clinicians make informed decisions regarding further cholesterol-lowering therapy in patients with T2DM on statin medications.

Although the exact mechanism is unknown, colesevelam has been shown to improve glycemic control in animal studies through its link to cholesterol gene regulation [49,50]. In human subjects, a smaller, double-blind study showed treatment with colesevelam did not affect GLP-1 secretion levels nor other plasma concentrations for glucose, insulin, or C-peptide [49]. In a larger, open-label study, colesevelam was shown to significantly decrease LDL-C and HbA1C levels in patients on stable statin and diabetic regimens. Future studies may further elucidate the actions of colesevelam on T2DM control. Currently, clinicians should evaluate their patient's needs and consider these findings with colesevelam treatment. As shown in Table 5, the recent clinical findings for colesevelam showed evidence for a greater efficacy at lowering HbA1C when compared with other BAS.

**Table 5:** Comparison of colesevelam with other common T2DM medications. Bajaj et al. compared colesevelam with ezetimibe over 24 weeks. Hansen et al. compared colesevelam with CDCA, among other drugs, and collected data upon drug installation (*=P<0.05). LDL-C: low-density lipoprotein-cholesterol; LOCF: last observation carried forward; GLP-1: glucagon-like peptide-1; CDCA: chenodeoxycholic acid; T2DM: type II diabetes mellitus

		LDL-C reduction from baseline at LOCF (%)	Glycated hemoglobin (HbA1C)reduction from baseline at LOCF (%)	Basal GLP-1 (pmol/L)	Basal glucose (mmol/L)
Bajaj et al. [51]	Colesevelam	14.0%	-0.26%	-	-
	Ezetimibe	23.2%*	-0.02%	-	-
Hansen et al. [48]	Colesevelam	-	-	11.3±1.8	9.6±0.9*
	CDCA	-	-	9.7±1.6	9.4±0.7*

## Conclusions

The ever-perpetuating burden of T2DM among those diagnosed has led to many recent critical discoveries that have transformed treatment protocols. More recently, the use of insulin as a treatment option for T2DM has decreased due to the development of newer non-insulin drug compounds. The development of new medications has provided an abundance of treatment options. However, many common treatment options can be costly and inconvenient for patients, leading to decreased adherence to therapies. Untreated T2DM is known to have accelerated disease outcomes, leading to the development of life-threatening complications. Cardiovascular disease accounts for the majority of diabetes-related mortalities in the United States. Providing treatment options that specifically target common complications and providing better adherence outcomes are two important objectives that require clinicians and researchers to adopt unconventional mechanisms for treating T2DM.

Adopting novel antidiabetic options from known safe medications used for treating other diseases is an effective and efficient way to increase T2DM treatment options. Currently, evidence of the efficacy of BC and colesevelam hydrochloride shows that both can successfully provide glycemic benefits by lowering common diabetes markers such as HbA1C. Furthermore, BC's effect on vascular tone allows this medication to potentially provide individuals protection against cardiovascular events by slowing down the progression of these complications. However, further studies should investigate these findings since the studies are limited. While the evidence for colesevelam hydrochloride's role in lowering cardiovascular disease is lacking, it has been postulated that its ability to lower blood LDL levels may translate into its ability to establish cardiovascular protection. Future studies should further explore the mechanisms of action for these drugs to provide a better understanding of the role they play in treating T2DM. Currently, literature on these medications is lacking, and more current studies should be conducted in a population of human participants rather than animal models. Future studies should also seek to ensure that sample sizes are realistic and contain the proper statistical power. Lastly, studying populations that are more generalizable would also provide a more effective means of indicating the efficacy of these medications.
